# Global transcriptome dynamics of seagrass flowering and seed development process: insights from the iconic seagrass *Zostera marina* L

**DOI:** 10.3389/fpls.2025.1545658

**Published:** 2025-03-13

**Authors:** Yu Zhang, Shidong Yue, Xinhua Wang, Mingjie Liu, Shaochun Xu, Xiaomei Zhang, Yi Zhou

**Affiliations:** ^1^ Chinese Academy of Sciences (CAS) Key Laboratory of Marine Ecology and Environmental Sciences, Institute of Oceanology, Chinese Academy of Sciences, Qingdao, China; ^2^ Laboratory for Marine Ecology and Environmental Science, Qingdao Marine Science and Technology Center, Qingdao, China; ^3^ Field Scientific Observation and Research Station of Yellow-Bohai Sea Temperate Seagrass Bed Ecosystems, Ministry of Natural Resources, Qingdao, China; ^4^ University of Chinese Academy of Sciences, Beijing, China

**Keywords:** seagrass, *Zostera marina*, sexual reproduction, flowering process, seed development, comparative transcriptomics

## Abstract

Seagrasses are the only group of higher angiosperms capable of fully living in seawater, playing a significant role in plant evolutionary history. However, studies on the molecular regulatory networks underlying sexual reproduction in seagrasses remain limited. This study evaluated the morphological changes of the spathe during eelgrass sexual reproduction and analyzed global transcriptome dynamics across eight sequential stages. The key findings are as follows:(1) Key flowering integrators such as FT, SOC1, AP1, and LFY exhibited high expression levels during the early stages, indicating their involvement in the induction of eelgrass flowering, consistent with terrestrial plants. (2) Based on the classical model of floral organ development in terrestrial plants – the “ABCDE model, genes related to the development of stamens, carpels, and ovules of eelgrass, including B-, C-, D-, and E-class genes, were identified. (3) Photosynthesis was temporarily suppressed after the initiation of sexual reproduction, and gradually resumed during the seed development stage, suggesting that the developed seed may perform photosynthesis. The Fv/Fm value (0.641 ± 0.028) of seeds at the developed seed stage further indicated that these seeds are indeed capable of photosynthesis. These findings provide important insights into the potential mechanisms underlying seagrass sexual reproduction and enrich knowledge of its reproductive genetics.

## 1 Introduction

About 100 million years ago, seagrasses returned to the ocean from land, readapting to the marine environment (Larkum et al, 2018). Currently, are the only flowering plants fully adapted to marine life (with only over 70 known species worldwide, far fewer than terrestrial plants), characterized by differentiated roots, stems, and leaves, capable of flowering and producing seeds, making them evolutionarily significant (Short et al., 2011; Olsen et al., 2016; Ma et al., 2024). Large, contiguous areas of seagrass are referred to as seagrass meadows, which provide highly valuable ecological services, including carbon sequestration, nutrient cycling, supporting fishery resources, and protecting coastlines against erosion (Fourqurean et al., 2012; Unsworth et al., 2019, Unsworth et al., 2022; Neto et al., 2022).

Sexual reproduction is the critical strategy for seagrasses to maintain long-term population stability under dynamic conditions. Studies indicate that sexual reproduction in seagrasses occurs underwater (hydrophily), with fully submerged male and female flowers that have simplified floral structures, often with reduced or absent sepals and petals, likely representing an adaptation to hydrophilous (primarily abiotic) pollination (Ackerman, 2006). Currently, there is a relatively good understanding of the morphology and phenology of sexual reproduction in seagrasses, but research on the molecular regulatory networks underlying sexual reproduction remains scarce. There is very limited research on flowering-related genes in seagrasses, with only a few articles reported. Entrambasaguas et al. (2017) was the first to apply *de novo* assembled transcriptome technology in seagrasses and identified a large number of genes and transcription factors related to flower development and flowering time in *Posidonia oceanica*. Marín-Guirao et al. (2019) studied the changes of genes expression related to flowering induction and flower organ development under heat stress conditions of *P. oceanica*. Ma et al. (2024) briefly explored the flowering organ genes in *Zostera marina* and *P. oceanica* based on genome assemblies. Apart from these studies, there are no other reports on the expression regulation of flowering-related genes in seagrasses.

*Zostera marina* L., also known as common eelgrass, belongs to the family Zosteraceae and the genus *Zostera*, which is the most widely distributed seagrass species on Earth (Moore and Short, 2006). Research on its flowering process has primarily focused on macroecology, while studies on the molecular regulatory mechanisms are almost nonexistent, except for the work mentioned above by Ma et al. (2024). Existing ecological studies have found that: eelgrass is monoecious, with flowers clustered in spathes located on branches of elongated stems that float vertically in the water column (Kuo and den Hartog, 2001; Walker et al, 2001); male and female flowers appear within the same spathe: the anthers emerge first, then filiform pollen is released in linear strands into the water column, floating within and between the seagrass meadows; following the transfer of pollen to stigma, the pollen germinates and a pollen tube grows through the stigma, style, and locule to the micropyle of the ovule where it penetrates the synergid cells; the pollen tube facilitates the transfer of sperm cells that fertilize the egg cell and the endosperm nucleus; after fertilization, the seeds develop within the spadices (Ackerman, 2006; Moore and Short, 2006). However, there have been no studies reporting the changes in gene expression during the continuous process of *Z.marina* flowering.

To sum up, the aim of this study is to explore which key genes and biological processes play crucial roles in flowering induction and flower organ development during the sexual reproduction of *Z. marina*., by conducting transcriptome analysis. Additionally, as the only group of higher angiosperms that lives exclusively in seawater, what are the similarities and differences in these key genes and biological processes compared to terrestrial plants? This study will provide a reference for further exploration of the flowering mechanisms in eelgrass. Moreover, the results offer an important basis for future research into the underlying mechanisms of seagrass flowering process and contribute to the enrichment of reproductive genetics knowledge in seagrasses.

## 2 Materials and methods

### 2.1 Collection of plant samples

**Figure 1:**
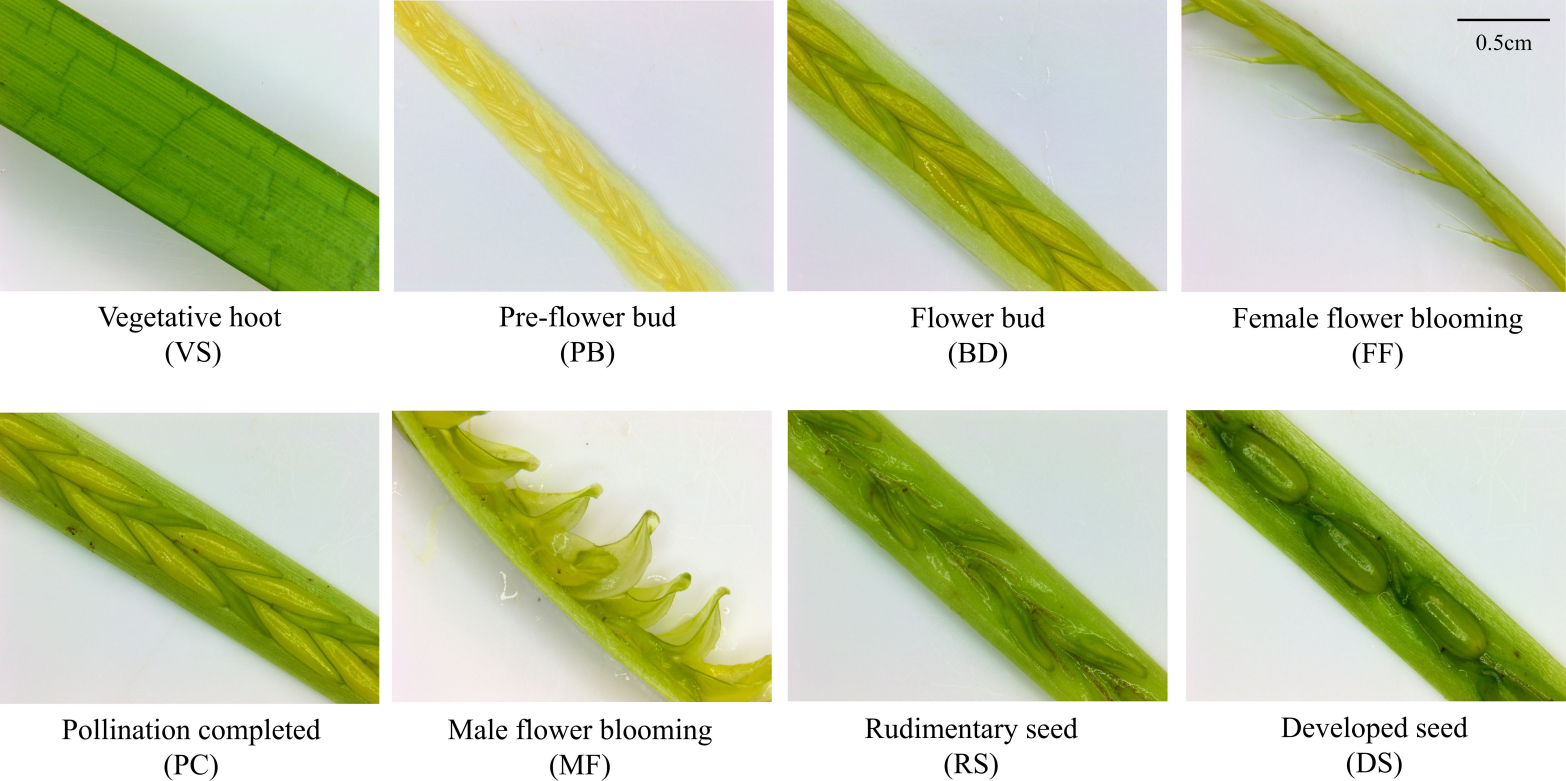
Vegetative shoot and seven stages of spathe development in eelgrass. Vegetative shoot (VS): The plant is in the vegetative growth stage, with intact and green leaves, and no floral organ differentiation. Pre-flower bud (PB): The flower bud begins to form, exhibiting a compact structure and a slightly pale yellow color. Flower bud (BD): The inflorescence continues to develop, displaying a more distinct structure, but it remains unopened. Female flower blooming (FF): The female flower starts to bloom, with the stigma becoming prominent and ready for pollen reception. Pollination completed (PC): Pollination is complete, and the two stigmas have fused together, while the male flower has not yet opened (Note: In the sampling process of this stage, pollination and fertilization cannot be accurately distinguished, so this stage may include both pollination and fertilization). Male flower blooming (MF): The stamens extend, and the anthers dehisce, releasing filamentous pollen into the water column. Rudimentary seed (RS): Following fertilization, the embryo begins to develop, and the seed structure starts to form but remains relatively small. Developed seed (DS): The seed has fully matured, with a well-defined structure.

In May 2023, eelgrass reproductive and vegetative shoots were collected in Qingdao Bay, Shandong Province (36°3′N, 120°19′E). The average water temperature during the collection period was recorded 16.15 ± 2.68°C, with a salinity of 32.5. The morphological changes during spathe development were assessed and were defined into seven morphologically distinct and sequential stages: pre-flower buds (PB), flower buds (BD), female flowers blooming (FF), pollination completed (PC), male flowers blooming (MF), rudimentary seed (RS), and developed seed (DS) (**Figure 1**). A total of 24 samples were collected, encompassing 8 stages of both vegetative and reproductive shoots (VS/PB/BD/FF/MF/PC/RS/DS, with 3 replicates for each stage). After washing all samples with distilled water, they were flash-frozen in liquid nitrogen for twenty minutes, then stored in a -80°C ultra-low temperature freezer for subsequent transcriptome analysis.

### 2.2 RNA extraction and sequencing

Total RNA from eelgrass tissues was extracted using the TRIzol (Majorbio, Shanghai, China) method, followed by DNase I (Cwbio, Jiangsu, China) treatment to remove genomic DNA. The concentration and purity of the extracted RNA were assessed using Nanodrop2000, while RNA integrity was evaluated via agarose gel electrophoresis and the RNA integrity number (RIN) value determined using Agilent2100. For a single library preparation, RNA quantity required was ≥1ug, concentration ≥ 35ng/μL, RIN ≥ 6.5, OD260/280 ≥ 1.8, and OD260/230 ≥ 1.0. Using magnetic beads coated with Oligo(dT) to pair with polyA tails through A-T base pairing, mRNA was isolated from total RNA. Next, the mRNA was fragmented using fragmentation buffer, and approximately 300bp fragments were selectively isolated through magnetic bead separation. Under the action of reverse transcriptase and random hexamers with six bases, a single-stranded cDNA was synthesized from the mRNA template, followed by second-strand synthesis to form a stable double-stranded structure. Then, the ends were repaired using end repair mix to create blunt ends, and an ‘A’ base was added at the 3’ end for the attachment of Y-shaped adapters. Subsequently, library enrichment was conducted, and after 15 cycles of PCR amplification, target bands of 200–300 bp were recovered from a 2% agarose gel. Following quantification using TBS380 (Picogreen), the samples were pooled according to data proportions and prepared for sequencing. Subsequent bridge PCR amplification was performed on the cBot to generate clusters, and finally, high-throughput sequencing was carried out on the Illumina NovaSeq 6000 platform (paired-end library, read length was 2×150bp).

### 2.3 Quality control and analysis of sequencing data

Clean data (reads) were obtained by filtering the raw data. These were aligned to the reference genome (source of reference genome: https://phytozome-next.jgi.doe.gov/info/Zmarina_v3_1) to generate mapped data (reads) for subsequent tasks such as transcriptome assembly and expression quantification. Simultaneously, quality assessment was performed on the alignment results of this transcriptome sequencing, focusing on sequencing saturation, gene coverage, distribution of reads across different regions of the reference genome, and reads distribution across different chromosomes. The software RSEM (Li and Dewey, 2011) (http://deweylab.github.io/RSEM/) was used for quantitative analysis of gene and transcript expression levels, quantified as TPM (transcripts per million reads). This quantification was carried out to facilitate the analysis of differential gene/transcript expression between different samples. To identify differentially expressed genes (DEGs) between two distinct samples, the software DESeq2 (Love et al., 2014) (http://bioconductor.org/packages/stats/bioc/DESeq2/) was utilized for gene differential expression analysis. The criteria for filtering significantly differentially expressed genes (DEGs) were set as follows: FDR < 0.05 & |log2FC| ≥ 1. The genes were compared against six major databases (NR, Swiss-Prot, Pfam, EggNOG, GO, and KEGG) to comprehensively obtain gene annotation information. KEGG pathway functional-enrichment analysis were carried out by KOBAS (http://kobas.cbi.pku.edu.cn/home.do). Principal component analysis (PCA), Venn analysis, DEG count statistics, heatmap analysis, Weighted Gene Co-expression Network Analysis (WGCNA), and Short Time-series Expression Miner (STEM) analysis were all performed on online platform of Majorbio Cloud Platform (www.majorbio.com).

### 2.4 Validation of RT-qPCR

Eight genes were selected randomly for validation of transcriptome using real-time quantitative polymerase chain reaction (RT-qPCR). Extensive studies have showed that the expression of 18S rRNA is stable across various organisms, tissues and developmental stages (Aman et al., 2012; Jarosova and Kundu, 2010; Tian et al., 2016; Weijn et al., 2013; Yu et al., 2021). And numerous seagrass transcriptome-related articles selected a single stable reference gene for transcriptome sequencing validation (Bruno et al., 2010; Chen and Qiu, 2020; Lin et al., 2018; Lv et al., 2018; Winters et al., 2011). So 18S rRNA were served as the internal reference for this research. Primer sequences are listed in the **Supplementary Table S1**. The RNA extracted earlier was used as a template for reverse transcription to obtain cDNA. Preliminary results indicated that each primer displayed a single bright band under specific conditions on agarose gel electrophoresis, suggesting no non-specific amplification, indicating suitability of the primers for subsequent experiments. The RT-qPCR reaction system comprised: 10μl of 2× ChamQ SYBR Color qPCR Master Mix, 0.8μl each of forward and reverse primers (at 5 μM each), 0.4μl of 50× ROX reference dye, 2μl of template (cDNA), and 6μl of ddH2O, making a total volume of 20μl. The cycling conditions for RT-qPCR were as follows: an initial step at 95°C for 5 minutes, followed by 40 cycles (denaturation at 95°C for 5 seconds, annealing at 55°C for 30 seconds, extension at 72°C for 40 seconds). Each treatment group consisted of 3 replicates, and each replicate sample had 3 technical replicates. Relative expression levels were calculated using the 2^−ΔΔCt^ method.

### 2.5 Assessment of seed photosynthetic capacity

Ten developed seeds were collected from the spathe at the DS stage for measuring the maximum potential photochemical efficiency of PSII (Fv/Fm), a chlorophyll fluorescence parameter widely used to assess plant photosynthetic capacity, by using the Diving PAM (Walz, Bavaria, Germany). Before measuring Fv/Fm, the seeds need to be placed under dark adaptation for 15–30 minutes.

## 3 Results

### 3.1 Comparative analysis of adjacent developmental stages

Transcriptome analysis was conducted on a total of 24 samples, encompassing 8 stages of both vegetative and reproductive shoots (VS/PB/BD/FF/MF/PC/RS/DS, with 3 replicates for each stage), resulting in 212.34 Gb of clean data. Each sample’s clean data exceeded 7.24 Gb, with a Q30 base percentage above 94.99%; the sequence alignment with the reference genome showed alignment rates ranging from 93.47% to 95.82%. PCA analysis results indicated that samples within the same group cluster together (**Supplementary Figure S1A**), while a noticeable difference was observed between different groups (excluding RS and DS groups, which overlap considerably). And it was evident that Principal Component 1 distinctly separated the VS group from the other 7 groups. Venn analysis reveals that among the 8 stages, there are 9852 genes shared among the samples, while the respective unique gene counts for each stage are 135, 308, 102, 93, 100, 79, 117, and 132 (**Supplementary Figure S1B**). The number of DEGs between adjacent stages are shown in the **Supplementary Figure S1C**. The RT-qPCR results corroborated the trends in gene expression observed in the RNA sequencing data, validating the reliability of the transcriptome sequencing (**Supplementary Figure S2**). To specifically understand the stepwise changes in key physiological processes during the sexual reproduction of eelgrass, we conducted a detailed pathways enriched analysis of the upregulated and downregulated DEGs sets between adjacent stages. The following section will focus on the stages PB vs. VS, BD vs. PB, PC vs FF, and RS vs. MF, where significant physiological changes occurred.

**Figure 2:**
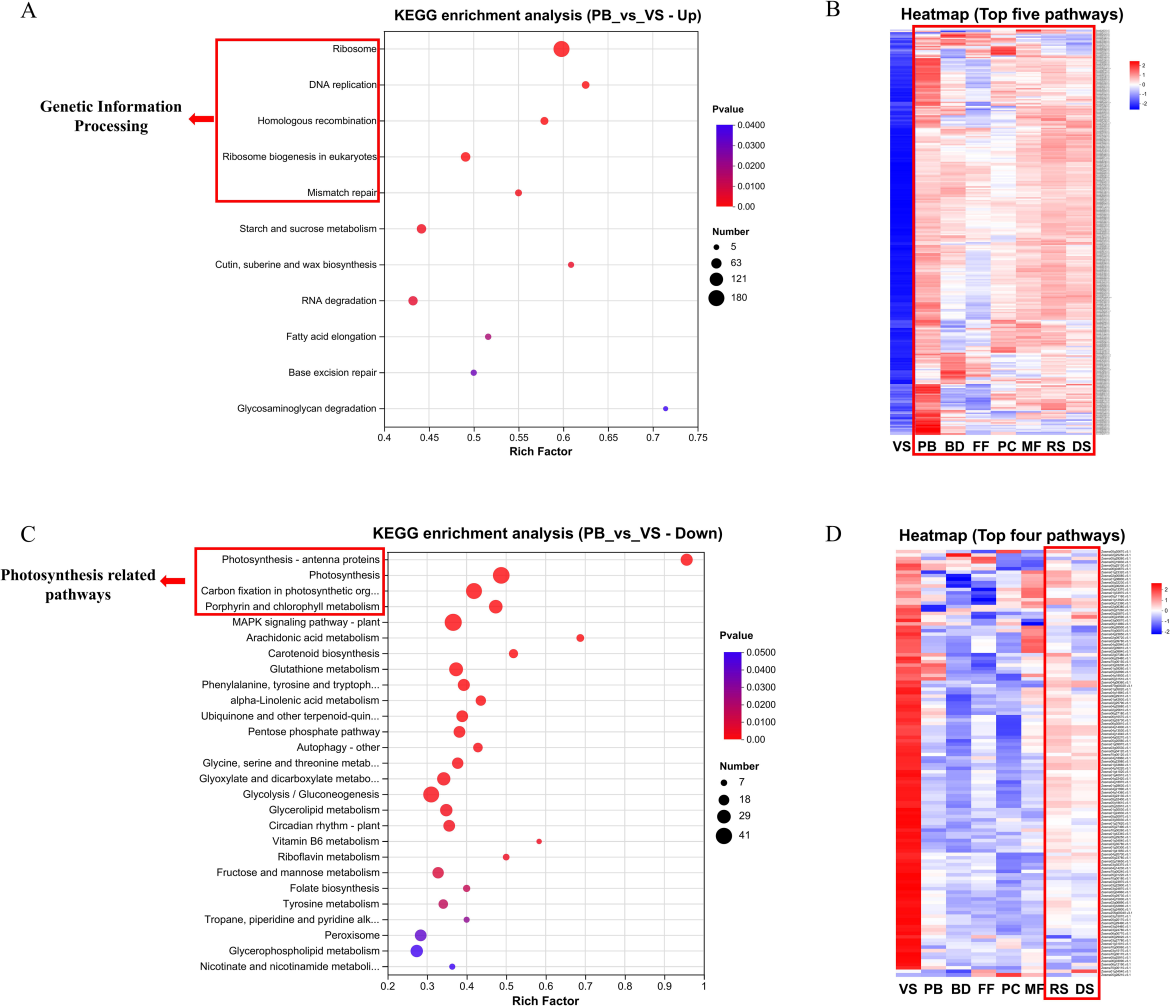
**(A)** KEGG enrichment analysis of PB vs. VS up-regulated DEGs set. **(B)** Heat map analysis of DEGs in first 5 significantly enriched genetic information processing-related pathways. **(C)** KEGG enrichment analysis of PB vs. VS down-regulated DEGs set. **(D)** Heat map analysis of DEGs in first 4 significantly enriched photosynthesis-related pathways. **(A, B)** The y-axis represents pathway names, while the x-axis indicates the rich factor. A larger rich factor represents a higher degree of enrichment. The size of the dot indicates the number of genes in the pathway, and the color of the dot corresponds to different p-value ranges. **(C, D)** Each column represents average gene expression level of three biological replicates for a specific stage, and each row represents a gene. The color indicates the normalized expression level of the gene in each sample. Red represents higher expression, while blue indicates lower expression. The specific range and trend of expression levels can be found in the numeric scale next to the color bar in the top right corner.

Compared to the VS stage, the top five significantly enriched pathways in the upregulated DEGs set of the PB stage are all related to Genetic Information Processing (**Figure 2A**). Heatmap analysis of DEGs enriched in these five pathways indicated that these genes are actively mobilized across the entire sexual reproduction phase (**Figure 2B**). In contrast, the top four significantly enriched pathways in the downregulated DEGs set of the PB stage, compared to the VS stage, are all related to photosynthesis (**Figure 2C**). Heatmap analysis of DEGs enriched in these four pathways indicated that photosynthesis begins to decrease in the PB stage and gradually recovers during seed formation (**Figure 2D**). The maximum potential photochemical efficiency of PSII (Fv/Fm) in DS stage seeds were also measured by using a Diving-PAM (WALZ, German), and the result showed a value of 0.641 ± 0.028 (N=10).

Compared to the PB stage, KEGG enrichment analysis of the upregulated DEGs set in the BD stage revealed that oxidative phosphorylation (map00190) is the most significantly enriched pathway and involves the largest number of genes among all pathways. Further visualization shows that this process is activated in both the BD and FF stages, while its expression levels are relatively low in the preceding and subsequent stages (**Supplementary Figure S3A**). The top four significantly enriched pathways in the downregulated DEGs set of the BD stage are all related to Genetic Information Processing, indicating a slight decrease compared to the previous stage.

Compared to the FF stage, KEGG enrichment analysis of the upregulated DEGs in the PC stage revealed that the Plant-pathogen interaction pathway (map04626) is the most significantly enriched pathway and contains the largest number of enriched DEGs. Heatmap analysis shows that it is noticeably activated starting from the PC stage (**Supplementary Figure S3B**). For the downregulated DEGs set, KEGG enrichment results indicate that oxidative phosphorylation (map00190) is the pathway with the highest number of enriched genes, totaling 63. This finding is consistent with results observed in the BD vs. PB comparison section.

Compared to the MF stage, KEGG enrichment analysis of the upregulated DEGs set in the RS stage revealed that four photosynthesis-related pathways were significantly enriched: Photosynthesis - antenna proteins (map00196), Carbon fixation in photosynthetic organisms (map00710), Photosynthesis (map00195), and Porphyrin and chlorophyll metabolism (map00860), collectively involving 46 DEGs. This result aligns with section PB vs. VS, indicating that photosynthesis becomes reactivated starting from this stage.

### 3.2 WGCNA analysis and STEM analysis

**Figure 3:**
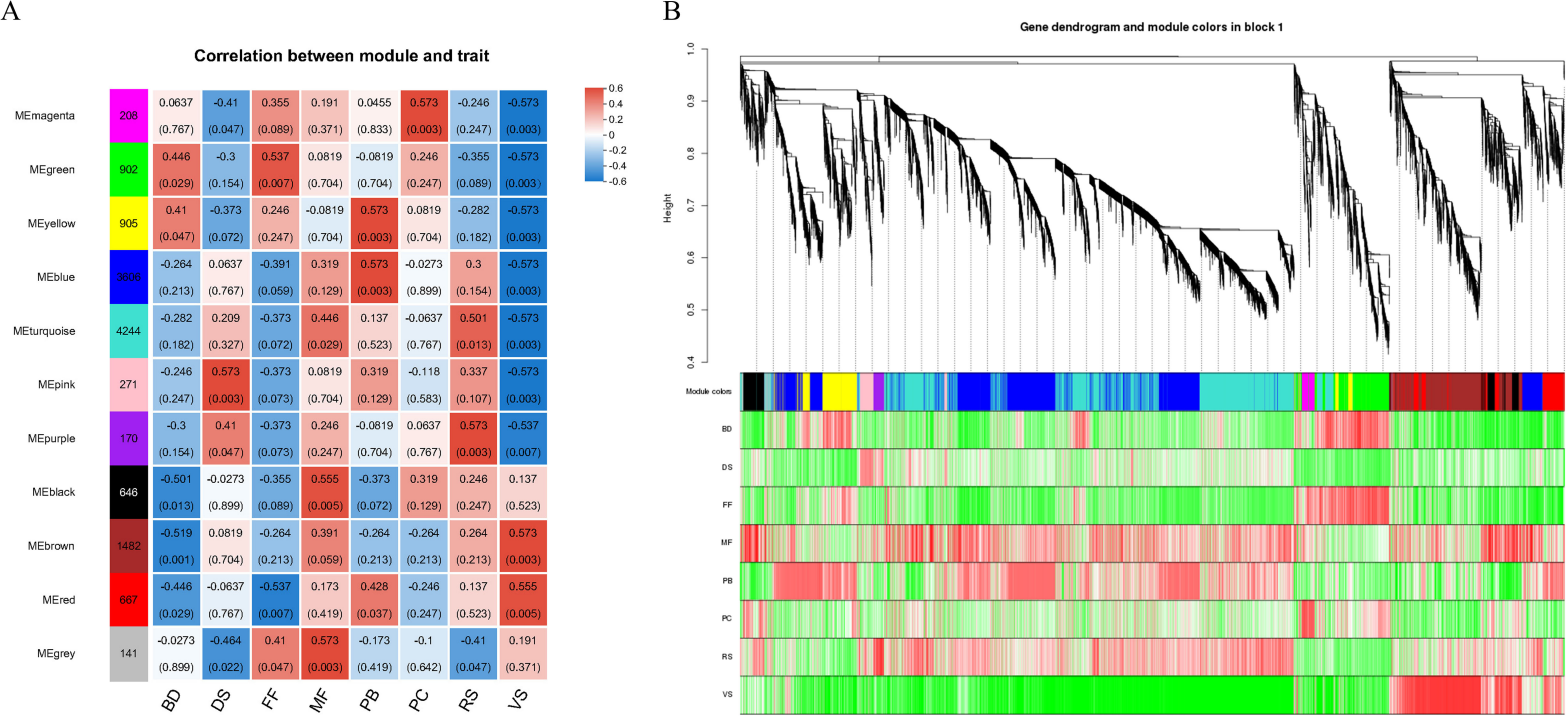
**(A)** Heat map of the correlation between modules and phenotypes. A column of numbers on the left represents the number of genes in the module, and data on the right represents the correlation coefficient and significance *p* value between the module and the phenotype (in parentheses). Blue indicates the negative correlation, and red indicates the positive correlation. **(B)** Heat map of gene and phenotype correlation. The upper part of the picture is a gene-level clustering tree, the middle part is the module to which the gene belongs, and the lower part represents the heat map of gene and trait correlation within the module. Red indicates the positive correlation, and green indicates the negative correlation.

Weighted Gene Co-expression Network Analysis (WGCNA) were conducted on all genes to identify highly correlated modules associated with flowering and seed development stages. The sample clustering analysis indicated that there are no outlier samples among the samples from these 8 stages. In total, 11 modules were identified, with the turquoise and blue modules containing the highest number of genes. Subsequently, the identified modules were associated with phenotype data to obtain key modules (**Figure 3A**). Simultaneously, the correlation between genes within the modules and the phenotype was specifically demonstrated (**Figure 3B**).

Modules that were significantly positively correlated with various stage were given focused attention: the VS stage is significantly associated with the brown and red modules (p<0.05); the PB stage is significantly associated with the yellow, blue, and red modules (p<0.05); the BD stage is significantly associated with the yellow and green modules (p<0.05); the FF stage is significantly associated with the green and gray modules (p<0.05); the PC stage is significantly associated with the magenta module (p<0.05); the MF stage is significantly associated with the gray, black, and turquoise modules (p<0.05); the RS stage is significantly associated with the purple and turquoise modules (p<0.05); and the DS stage is significantly associated with the pink and purple modules (p<0.05). Adjacent stages generally share at least one module that is significantly correlated. Genes from each stage’s significantly correlated modules were combined and subjected to KEGG pathway enrichment analysis to identify the key biological processes active in each stage.

In the VS stage, the top five significantly enriched pathways are all related to photosynthesis, with a total of 117 genes enriched. This stage also shows a high number of significantly enriched pathways related to metabolism, encompassing 252 genes in total. In the PB stage, the significantly enriched pathways are mainly associated with genetic information processing, with 400 genes enriched. The BD and FF stages have significantly enriched pathways primarily related to metabolism, with 85 genes and 61 genes enriched, respectively. In the PC stage, only one pathway is significantly enriched, which is DNA replication, with just 3 genes enriched. In the MF and RS stages, the significantly enriched pathways are also predominantly related to metabolism, with 271 and 178 genes enriched, respectively. Additionally, Ribosome (map03010) is the most significantly enriched pathway in both stages and has the highest number of enriched genes, with 185 genes in the MF stage and 184 genes in the RS stage. In the DS stage, all significantly enriched pathways are related to metabolic processes, with a total of 46 genes enriched.

Based on Short Time-series Expression Miner (STEM) analysis, we conducted a temporal analysis of samples across the 8 stages to explore gene expression patterns during the flowering and seed development processes of eelgrass at different time points (**Supplementary Figure S4**). The results showed that a total of 15 profiles exhibit significant temporal patterns (p<0.05, indicated by colored blocks), among which profile 49 displays an upward trend throughout the entire process. Further KEGG functional enrichment analysis of the gene set in profile 49 revealed 17 significantly enriched pathways (p<0.05), of which 16 pathways belong to the primary category of Metabolism. The Starch and sucrose metabolism pathway was the most significantly enriched and contained the largest number of genes.

### 3.3 Flowering pathways and floral organ development in eelgrass

**Figure 4:**
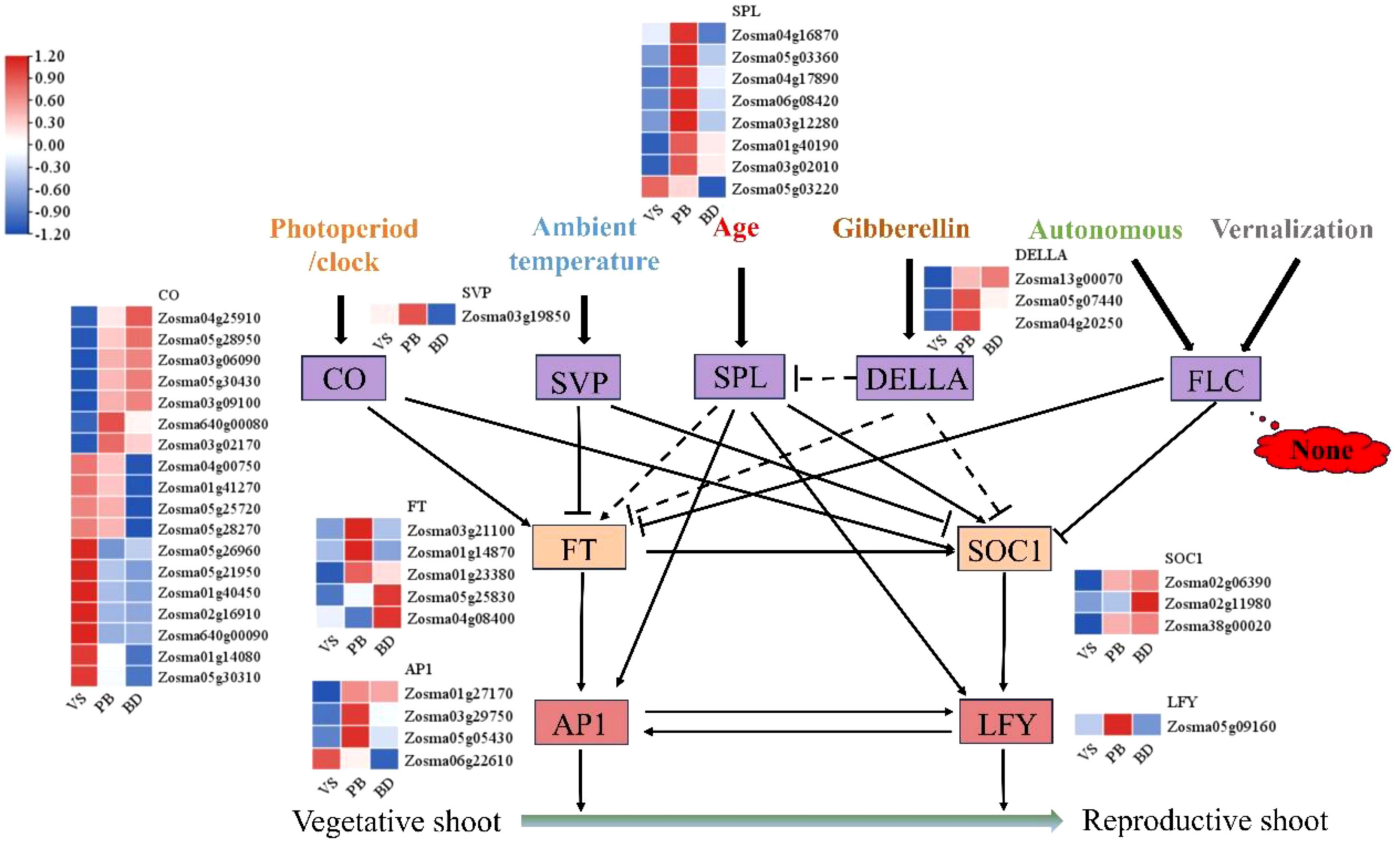
Heat map analysis of key genes in eelgrass potential flowering pathway. The purple boxes, including CO, SVP, SPL, DELLA, and FLC, indicate key regulators in different flowering pathways; the yellow boxes, including FT and SOC1, indicate important main floral integrators. The red boxes, including AP1 and LFY, which work together to promote the establishment of the floral meristem. Solid lines represent direct regulation, while dashed lines indicate indirect regulation. Arrows signify positive regulation, and T-shaped ends indicate negative regulation. This diagram only presents a selection of major regulatory genes, while the complete flowering-time gene networks involve over 300 genes, which can be explored in the Flowering-Interactive Database (Bouché et al., 2016).

Expression profiles of 9 key flowering integrators across the first three stages were presented (VS, PB, and BD) (**Figure 4**). A total of 18 CONSTANS-LIKE genes were identified, with 7 showing high expression in the PB and BD stages; 1 SHORT VEGETATIVE PHASE(SVP) gene, which was highly expressed in the PB stage; 3 DELLA proteins, of which 2 were highly expressed in the PB stage and 1 in the BD stage; 8 SQUAMOSA PROMOTER BINDING PROTEIN-LIKE(SPL) family genes, of which 7 were highly expressed in the PB stage; 5 FT genes, with 3 highly expressed in the PB stage and 2 in the BD stage; 3 SOC1 genes, all highly expressed in the BD stage; 4 APETALA1(AP1) genes, with 1 highly expressed in the VS stage and 3 in the PB stage; and 1 LFY gene, which was highly expressed in the PB stage. Notably, FLC gene expression was not detected throughout the entire flowering period.

**Figure 5:**
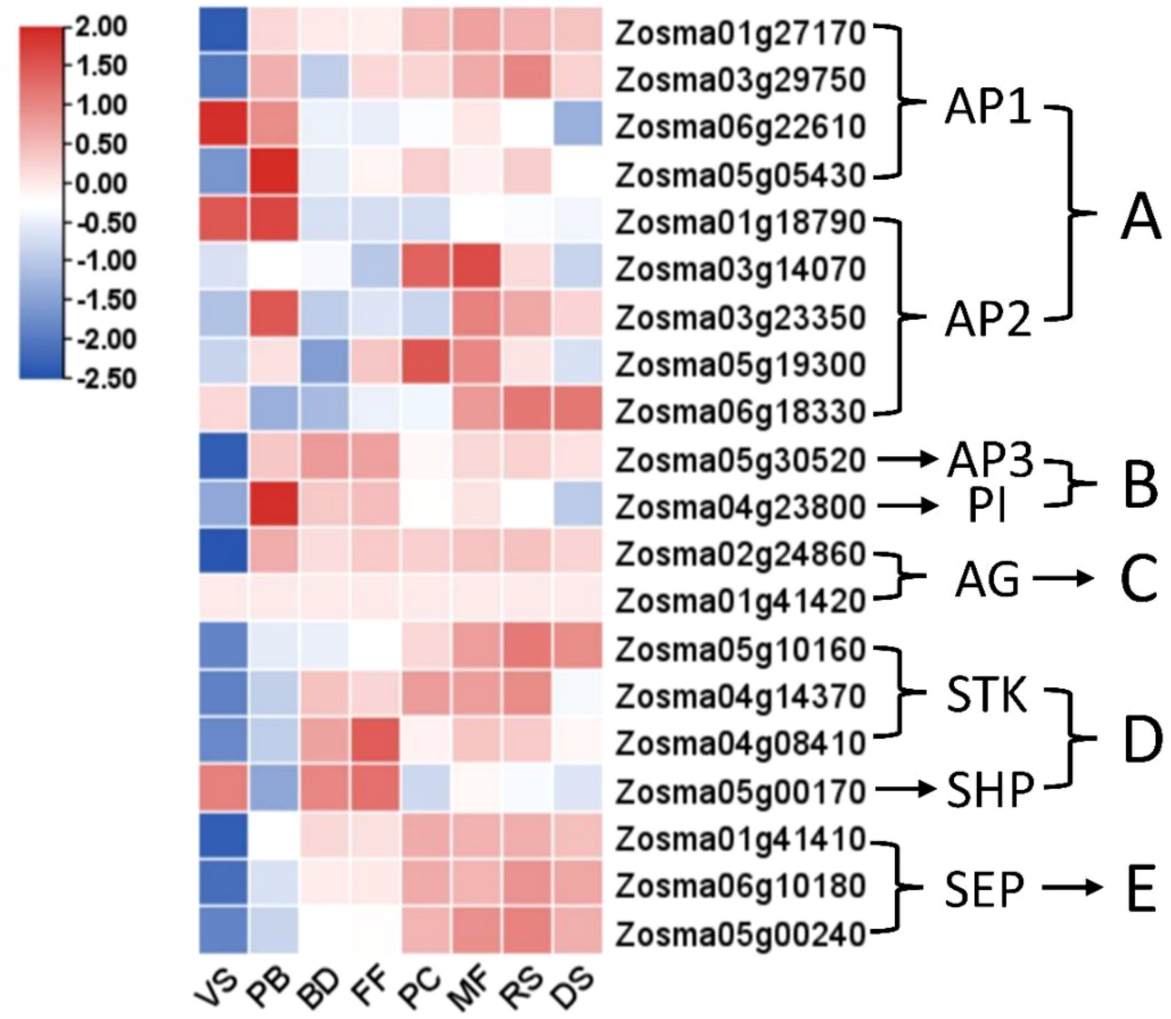
Heat map analysis of flower development-related genes belonging to the **(A–E)** family at various stages in eelgrass. Based on studies in terrestrial plants, stamens are primarily determined by B-class and C-class genes, while carpels are mainly determined by C-class and D-class genes, D-class genes also participate in ovule formation, E-class genes integrate the development of all floral organs. (The other legend is the same as in **Figures 2C, D**).

Expression profiles of ABCDE-related genes for floral organ development from the eelgrass transcriptome were presented in **Figure 5**, with the following results: 9 A-class genes with 4 for AP1 and 5 for APETALA2(AP2); 2 B-class genes with 1 for APETALA3(AP3) and 1 for PISTILLATA(PI); 2 C-class genes for AGAMOUS (AG); 4 D-class genes with 3 for SEEDSTICK(STK) and 1 for SHATTERPROOF(SHP); and 3 E-class genes for SEPALLATA(SEP). The A-class gene AP1 shows consistent expression across all stages, while AP2 is primarily highly expressed in the later stages. The B- and C-class genes exhibit high expression levels throughout all stages following the onset of flowering (PB). The D- and E-class genes show sustained high expression starting from the BD stage.

### 3.4 Analysis of hormone-related genes

Expression profiles of genes related to abscisic acid (ABA), gibberellins (GA), auxins (IAA), cytokinins (CK), jasmonic acid (JA), and salicylic acid (SA), were presented in the form of heatmaps (**Supplementary Figure S5**). A total of 20 ABA-related genes were identified, of which 17 are involved in ABA synthesis and positive regulation, while 3 are related to ABA degradation and negative regulation. 12 GA-related genes were identified, with 6 involved in GA synthesis and 6 in GA inactivation and negative regulation. A total of 98 auxin-related genes were identified, most of which are involved in IAA signal transduction. 14 CK-related genes were identified, with 7 involved in cytokinin synthesis and positive regulation, and 7 in degradation. 12 JA-related genes were identified, of which 8 are involved in JA synthesis and positive regulation, and 4 in JA negative regulation. Finally, 11 SA-related genes were identified, all of which are involved in SA synthesis and positive regulation.

## 4 Discussion

### 4.1 Key biological processes with significant changes during the sexual reproduction of eelgrass

It is well known that flowering is a complex physiological process that requires the involvement of numerous proteins to regulate the formation and development of floral organs. Our transcriptome data indicated that during the early stage of eelgrass flowering (PB stage), a large number of genes related to Genetic Information Processing were upregulated, particularly those involved in the ribosome pathway. Ribosome genes are traditionally considered housekeeping genes responsible for protein synthesis, translating genetic information from mRNA into proteins. During the flowering process, ribosomes play a key role by synthesizing crucial regulatory proteins and signal transduction molecules, thereby regulating flowering induction, inflorescence development, and floral organ formation (Degenhardt and Bonham-Smith, 2008; Stirnberg et al., 2012).

The differentiation and development of flower buds depend on the availability of carbohydrates, and a deficiency in carbohydrates can result in underdeveloped petals or the cessation of flower development (Wang et al., 2008; Önder et al., 2023). Our findings revealed that carbohydrate metabolism-related pathways, particularly the starch and sucrose metabolism pathways, are highly active throughout the flowering process of eelgrass, indicating their crucial role in eelgrass flowering. On the one hand, the oxidative metabolism of carbohydrates generates ATP, which provides energy for flower growth and opening (Atkin et al., 2000). On the other hand, carbohydrate metabolism supplies the necessary carbon sources for the growth and development of eelgrass, including monosaccharides such as glucose and fructose, and polysaccharides like sucrose (Yap et al., 2008). Consistent with this, we observed that the oxidative phosphorylation pathway exhibits high expression during the BD and FF stages. Oxidative phosphorylation is a key cellular process for energy production, generating ATP via the mitochondrial electron transport chain and ATP synthase (Wilson, 2017). Therefore, during the flowering process of eelgrass, oxidative phosphorylation likely plays a critical role in energy supply, with the flower bud stage and female flower opening stage being the periods of high energy production.

Our transcriptome results revealed that the Plant-pathogen interaction pathway (map04626) becomes significantly activated after the female flower opening stage (FF, **Supplementary Figure S3B**). This suggested that eelgrass may be more susceptible to pathogen invasion following the opening of female flowers. Pathogen infection could potentially affect the formation and development of floral organs, leading to abnormalities such as bud wilting or reduced pollen production, thereby disrupting the normal flowering process (Ngugi and Scherm, 2006; Andargie and Li, 2016). The activation of the Plant-pathogen interaction pathway enables eelgrass to detect pathogen invasion, rapidly transmit signals, activate a series of defense-related genes, and mount an effective immune response in a timely manner, like other reported plants (Oeser et al., 2009; Chao et al., 2014).

As flower development progresses, flowers transition from autotrophic to heterotrophic. Young green petals are capable of photosynthesis, but as they mature, they gradually lose their ability to fix carbon and eventually become fully heterotrophic (Thomas et al., 2003; Müller et al., 2010; Muhlemann et al., 2012). This is consistent with our transcriptome data, where we observed that several photosynthesis-related pathways were more active during the vegetative stage, but their activity declined in the early stage of sexual reproduction (PB stage), with related genes expression significantly downregulated and remaining low throughout the flowering period. Similarly, Ge et al. (2017) observed a downregulation in the expression of photosynthesis-related genes during the flowering process in bamboo. Based on this, we speculate that the carbohydrates needed for eelgrass flowering are likely sourced from photosynthetic products synthesized in other leaves and transported to the reproductive tissues. Studies by Eveland and Jackson (2012) and Borghi and Fernie (2017) also found that carbohydrates such as sucrose can indeed be translocated from photosynthetically active tissues to non-photosynthetic sinks, like flowers.

Moreover, we observed that during the seed development stages (RS and DS stages) after flowering, photosynthesis-related pathways and genes become active again, suggesting that developed seed may perform photosynthesis, Fv/Fm value (0.641 ± 0.028) of seeds at the DS stage further indicated that these seeds are indeed capable of photosynthesis. We hypothesize that this mechanism may be similar to that of seeds in some terrestrial plants: on one hand, photosynthetic products provide nutrients for the growing embryo, meeting the energy demands of seed development; on the other hand, the oxygen released during photosynthesis helps maintain oxygen levels within the seed to support adequate cellular respiration (Smolikova and Medvedev, 2016; Shackira et al., 2022).

### 4.2 Flowering pathways and floral organ development in eelgrass

The genetic network regulating flowering has been extensively studied in the model terrestrial plant *Arabidopsis thaliana*, but this knowledge remains limited in most other plant species and is especially lacking in seagrasses.

Almost all flowering pathways (including photoperiod, vernalization, ambient temperature, aging, gibberellin, and autonomous pathways) converge on an important integrator, FT, which encodes a protein known as “florigen.” Florigen promotes flowering by activating floral meristem identity genes and other flowering initiators (Corbesier et al., 2007; Khan et al., 2014). SOC1, a member of the MADS-box family, integrates multiple flowering regulatory pathways such as autonomous, vernalization, and aging, and positively regulates floral development genes (Jung et al., 2012). LFY and AP1 are two key floral meristem identity genes that function redundantly with additive effects, aiming to promote the growth of primordia into flowers rather than leaf buds at the flanks of the inflorescence meristem (William et al., 2004). Our transcriptome data identified a total of 5 FT genes, 3 SOC1 genes, 4 AP1 genes, and 1 LFY gene, all of which showed higher expression in the PB stage. This suggests that these genes play a role in the induction of flowering in eelgrass, consistent with the mechanisms observed in terrestrial plants (Leijten et al., 2018).

The CONSTANS (CO) gene regulates the photoperiod-induced flowering process in plants (Yoo et al., 2005). However, our transcriptome data did not identify the CO gene, but found 18 CO-like genes, 7 of which were highly expressed during the PB or BD stage of eelgrass. The SPL gene has been shown to play a crucial role in the flowering age pathway, as it can directly bind to the promoters of SOC1 and LFY, activating their transcription and regulating the initiation of the floral meristem (Wang et al., 2009; Xu et al., 2016). Our transcriptome data identified 8 genes from the SPL family, 7 of which were highly expressed during the PB stage of eelgrass, promoting the transition from vegetative to reproductive growth. DELLA proteins are known as flowering inhibitors in the gibberellin pathway (Galvao et al., 2012). However, contrary to our expectations, our transcriptome results revealed that the 3 DELLA proteins identified were highly expressed in the early stages of flowering. This suggests that the gibberellin pathway in eelgrass flowering may function through other genes rather than DELLA proteins. SVP is a central regulator in the ambient temperature flowering pathway, negatively regulating the FT gene (Li et al., 2008; Jang et al., 2009). Our transcriptome data identified only one SVP gene, which showed higher expression during the VS and PB stages, but decreased expression during the BD stage. This indicated that the downregulation of SVP expression during eelgrass flowering may relieve the repression on FT transcription, allowing flowering to proceed. FLC is a core gene in the vernalization and autonomous pathways, negatively regulating flowering by inhibiting FT and SOC1 expression (Sheldon et al., 2000; Wu et al., 2020). However, some plants can promote flowering via FLC-independent mechanisms in the vernalization pathway (Alexandre and Hennig, 2008. Our transcriptome results showed no existence of the FLC gene throughout the entire eelgrass flowering period, suggesting that the vernalization and autonomous pathways in eelgrass may operate through FLC-independent mechanisms.

To adapt to underwater pollination, eelgrass has avoided complex floral organs, resulting in relatively simple flower structures lacking typical petals and sepals. Its inflorescence is a spadix, usually enclosed by a large, flat spathe, with male and female flowers arranged along the main axis (Kuo and den Hartog, 2001; Moore and Short, 2006). Based on studies in terrestrial plants, stamens are primarily determined by B-class and C-class genes, while carpels are mainly determined by C-class and D-class genes (Theißen et al., 2016). In this study, two B-class genes (AP3, Zosma05g30520 and PI, Zosma04g23800) and one C-class gene (AG, Zosma02g24860) were highly expressed from the PB to FF stages, suggesting a foundational role in the subsequent formation and development of male flowers. One C-class gene (AG, Zosma02g24860) and four D-class genes (STK, Zosma05g10160/Zosma04g14370/Zosma04g08410 and SHP, Zosma05g00170) exhibited high expression from the initial flowering transition to the female flower blooming stage (from PB to PC), indicating their involvement in carpel formation and development. When mature carpel receives the pollen, it travels through the pollen tube on the stigma into the ovary, successfully reaching the ovule cell for fertilization (Ackerman et al., 2006). However, samples in PC stage in this research may include both the pollination and fertilization processes, so it is not yet possible to clearly identify the genes that play the major role in each of these processes separately. Additionally, studies have shown that D-class genes also participate in seed formation (Theißen et al., 2016). Consistent with this, we observed high expression of three STK genes (Zosma05g10160/Zosma04g14370/Zosma04g08410) from pollination completion to seed development stages (from PC to DS). According to previous research, E-class genes integrate the development of all floral organs (Theißen et al., 2016). Consistent with this, in our findings, three E-class genes (SEP, Zosma01g41410/Zosma06g10180/Zosma05g00240) mainly functioned from BD to DS stages.

It is noteworthy that although studies in terrestrial plants indicate that A-class genes (AP1 and AP2) primarily regulate sepal and petal development (Theißen et al., 2016), and such genes were indeed expressed in eelgrass transcriptome data, the simplified flower structure of eelgrass lacks petals and sepals. Thus, we hypothesize that AP1 and AP2 have other important roles in eelgrass sexual reproduction. For instance, AP1 may be involved in the formation of eelgrass inflorescence meristems, suppressing vegetative growth and initiating the flowering phase. Supporting this hypothesis is the observation that three AP1 genes (Zosma01g27170/Zosma03g29750/Zosma05g05430) exhibited low expression during the vegetative stage but were highly expressed at the onset of flowering (PB stage). Research also suggests that AP2 genes may participate in seed development (Ohto et al., 2005). Consistent with this, our transcriptome data revealed that four AP2 genes (Zosma03g14070/Zosma03g23350/Zosma05g19300/Zosma06g18330) exhibited high expression from pollination completion through seed development (from PC to DS stage), indicating a potential role in seed formation and development in eelgrass.

### 4.3 Changes in hormone-related genes during the sexual reproduction of eelgrass

Plant hormones, as key regulators of flowering time and floral development, have been confirmed in many plants (Davis, 2009). Abscisic acid (ABA), a sesquiterpenoid plant hormone, is involved in various growth and developmental processes, including seed storage protein and lipid synthesis, seed dormancy, and flowering (Trivedi et al., 2016). In this study, genes related to ABA synthesis and positive regulation were found to have high expression levels throughout the flowering and seed development stages, indicating that ABA plays an important role during the entire sexual reproduction phase of eelgrass.

Gibberellins (GA) are key hormones that influence plant development, flowering induction, and seed germination (Hedden and Sponsel, 2015). In this study, genes related to GA synthesis were found to be upregulated as flowering progressed, with the highest expression observed in the final DS stage. This indicates that the role of GA increases throughout eelgrass’s sexual reproduction phase and becomes more prominent during seed development.

Auxin is another important hormone involved in the flowering process, particularly in regulating the formation of inflorescences and floral organs (Cucinotta et al., 2021). Our transcriptome data identified a large number of genes associated with auxin signaling, including IAA, ARF, SAUR, AUX/IAA, AUX/LAX, and PIN protein genes. Overall, these genes showed higher expression levels during the PB stage and later stages, including PC, MF, RS, and DS stages. Based on existing research in terrestrial plants, we hypothesize that early activation of auxin signaling may be related to the formation of eelgrass inflorescences (Cucinotta et al., 2021); in the full-flowering stage, auxin signaling activation may be associated with pollen and anther maturation, development, and release in eelgrass (Cecchetti et al., 2008; Galbiati et al., 2013); during seed development, auxin signaling likely plays a role in the development of the embryo and seed coat after fertilization (Cao et al., 2020).

Cytokinins (CK) not only regulate cell division and differentiation in the floral meristem but are also essential for pollen development (Huang et al., 2003; Jacqmard et al., 2003). In this study, we found that genes related to CK synthesis and positive signaling were expressed throughout all stages, while CK degradation-related genes showed high expression starting from the PC stage and continuing through to the DS stage. This suggests that CK play a role in the early stages of eelgrass flowering. Similar findings were observed by other researchers, who noted that in the early stages of floral bud differentiation in lychee, cytokinin concentrations were high, and cytokinin biosynthesis genes were upregulated (Zhang et al., 2014).

Salicylic acid (SA) is a member of phenolic compounds characterized by an aromatic ring and hydroxyl or other functional groups (Raskin, 1992). Phenylalanine ammonia-lyase (PAL) controls SA synthesis and is involved in the flowering process of plants (Wada et al., 2014). NON-EXPRESSOR OF PR1 (NPR1) is a transcription coactivator that have important defensive functions during flowering and could also play a role in flowering regulation (Cuzick et al., 2008). Xu et al. (2019) found that MuNPR1 transgenic Arabidopsis exhibited an early-flowering phenotype. Our transcriptome results showed that both the PAL and NPR1 genes had high expression levels starting from the PC stage and continuing through to the DS stage in eelgrass. This suggests that SA plays a significant role in the later stages of eelgrass flowering and during seed development.

The synthesis and accumulation of jasmonic acid (JA) have been identified as critical steps in flower development during plant breeding (Wasternack et al., 2013). The JAR1 gene is an essential signaling gene required for JA-regulated flowering and anther dehiscence in rice (Xiao et al., 2014). In this study, genes related to JA synthesis (OPR and AOC) as well as the JAR1 gene showed high expression starting from the MF stage and continuing through to the DS stage in eelgrass, which indicates that JA plays a significant role during the male flower blooming process (i.e., anther dehiscence) and in subsequent seed development.

## 5 Conclusion

To our knowledge, the flowering and seed development dataset of eelgrass used in this study represents the most comprehensive dataset for detailed analysis of the dynamic genome-wide expression profiles during seagrass sexual reproduction. Through comparative analyses of differential gene expression between different spathe developmental stages and vegetative shoots, some key pathways and candidate genes significantly related to eelgrass flowering and seed development were identified. Furthermore, based on the flowering pathways and ABCDE model of floral organ development in terrestrial plants, key genes were highlighted in eelgrass, indicating a high degree of conservation in flowering mechanisms between seagrasses and terrestrial plants. Future studies should focus on elucidating the more specific molecular pathways involved in the various flowering pathways of *Z.marina*, as well as investigating the actual functional roles of ABCDE family genes in the development of its floral organs. The findings provide an important foundation for in-depth exploration of seagrass floral development mechanisms and enrich knowledge of its reproductive genetics.

## Data Availability

The datasets presented in this study can be found in online repositories. The names of the repository/repositories and accession number(s) can be found below: https://www.ncbi.nlm.nih.gov/, PRJNA1178179.
