# Supplementary material for: Global transcriptome dynamics of seagrass flowering and seed development process: insights from the iconic seagrass Zostera marina L
Source: Front Plant Sci. 2025 Mar 13;16:1545658. doi: 10.3389/fpls.2025.1545658 (PMC11965923; doi:10.3389/fpls.2025.1545658)
Supplement: Supplementary file 1 [file DataSheet1.docx]

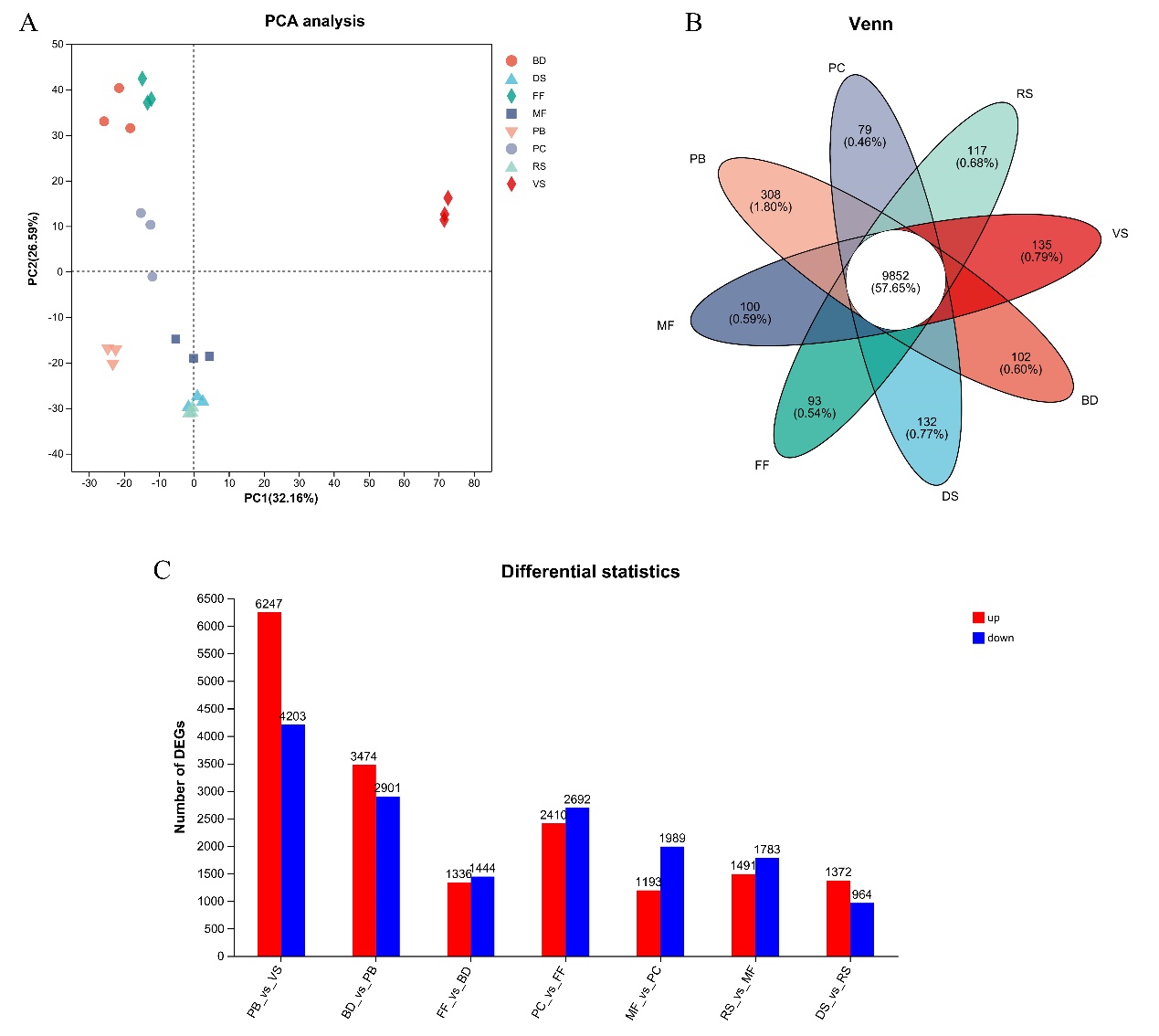


**Fig. S1 A. Principal component analysis. Eight different shapes correspond to the eight sampling stages, with three biological replicates for each stage. The distance between sample points represents the similarity between samples, where shorter distances indicate higher similarity.; B. Venn analysis. The central white circular region represents the genes commonly expressed across all eight stages, while the petal-shaped regions in different colors indicate stage-specific expressed genes. C. Statistics of the number of DEGs in adjacent phases. The x-axis represents different differential comparison groups. while the y-axis indicates the number of upregulated and downregulated genes. Red bars represent upregulated genes, and blue bars represent downregulated genes.**


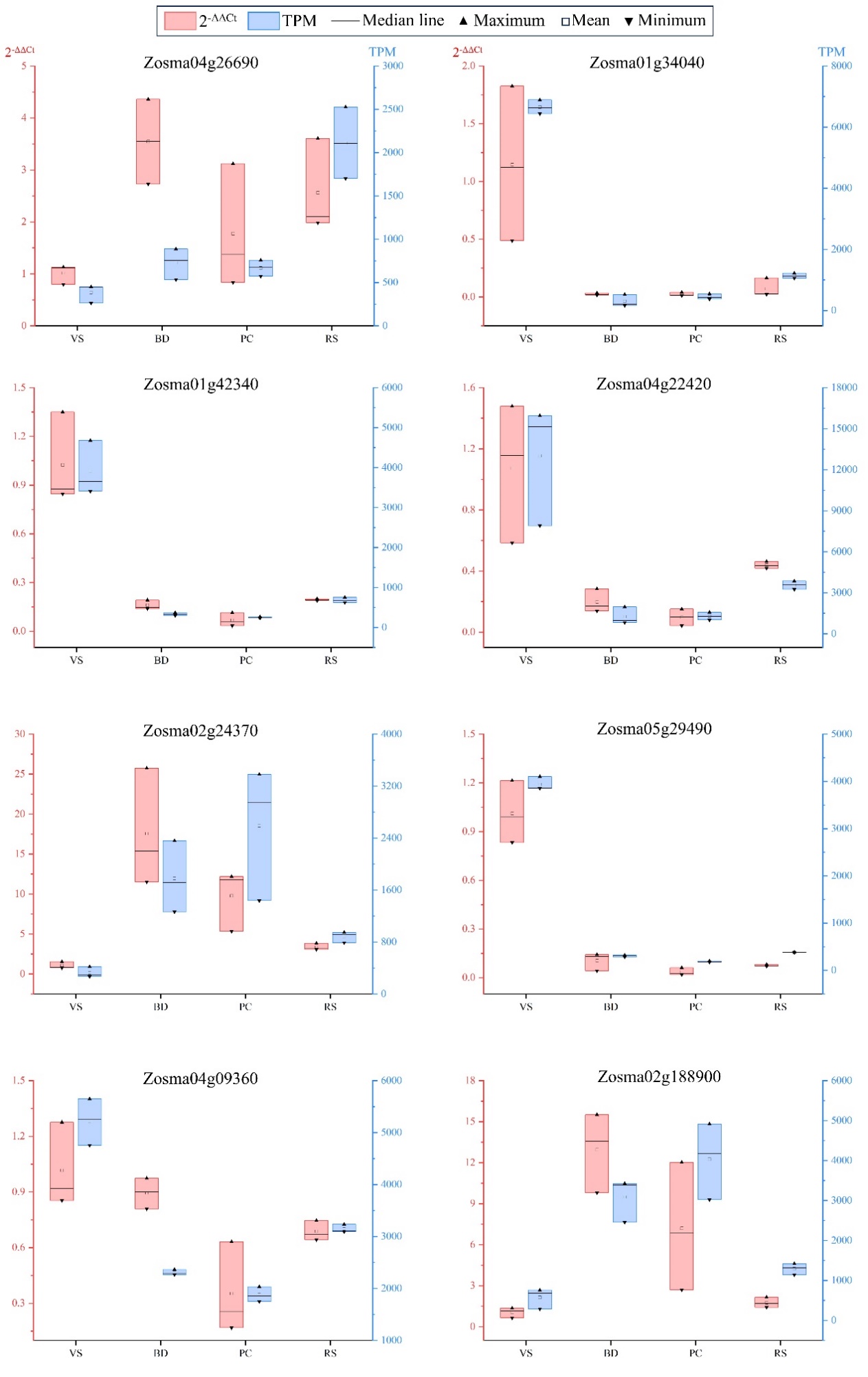


**Fig.S2 Validation of RNA-seq results by RT-qPCR.The red box plot (left Y-axis) represents the RT-qPCR results, indicating the relative gene expression levels (2^−ΔΔCt). The blue box plot (right Y-axis) represents the RNA-seq results, indicating gene expression abundance (TPM, transcripts per million). The black line represents the median value, the black triangle indicates the maximum value, the black inverted triangle indicates the minimum value, and the hollow square represents the mean value. The X-axis represents different developmental stages: VS：Vegetative Shoot stage; BD: Flower Bud stage；PC: Pollination Completed stage; RS: Rudimentary Seed stage.**


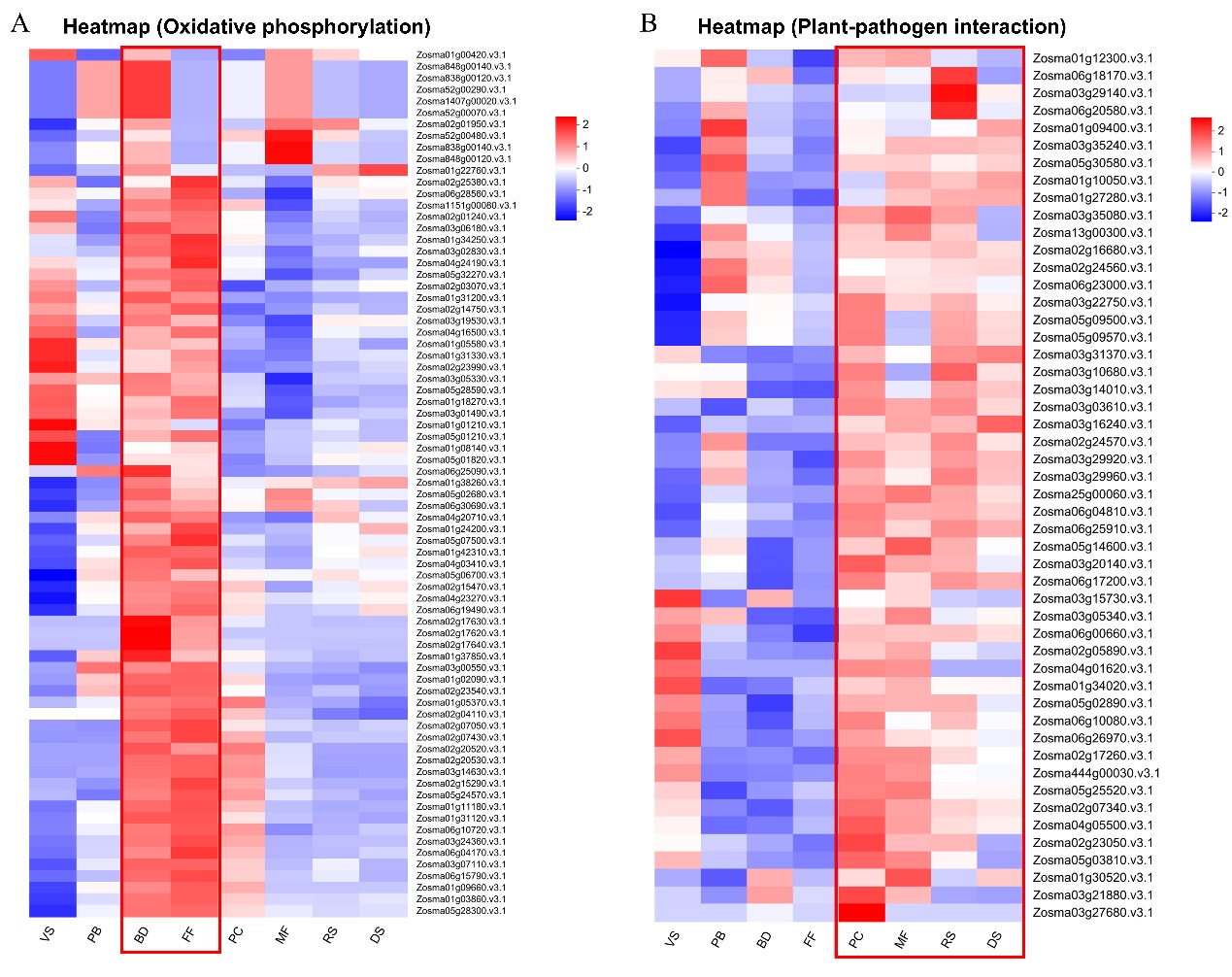


**Fig.S3 A. Heat map analysis of genes in oxidative phosphorylation pathway in upregulated DEGs set in BD vs. PB. B. Heat map analysis of genes in plant-pathogen interaction pathway in upregulated DEGs set in PC vs. FF.** **The color indicates the normalized expression level of the gene in each sample. Red represents higher expression, while blue indicates lower expression. The specific range and trend of expression levels can be found in the numeric scale next to the color bar in the top right corner.**


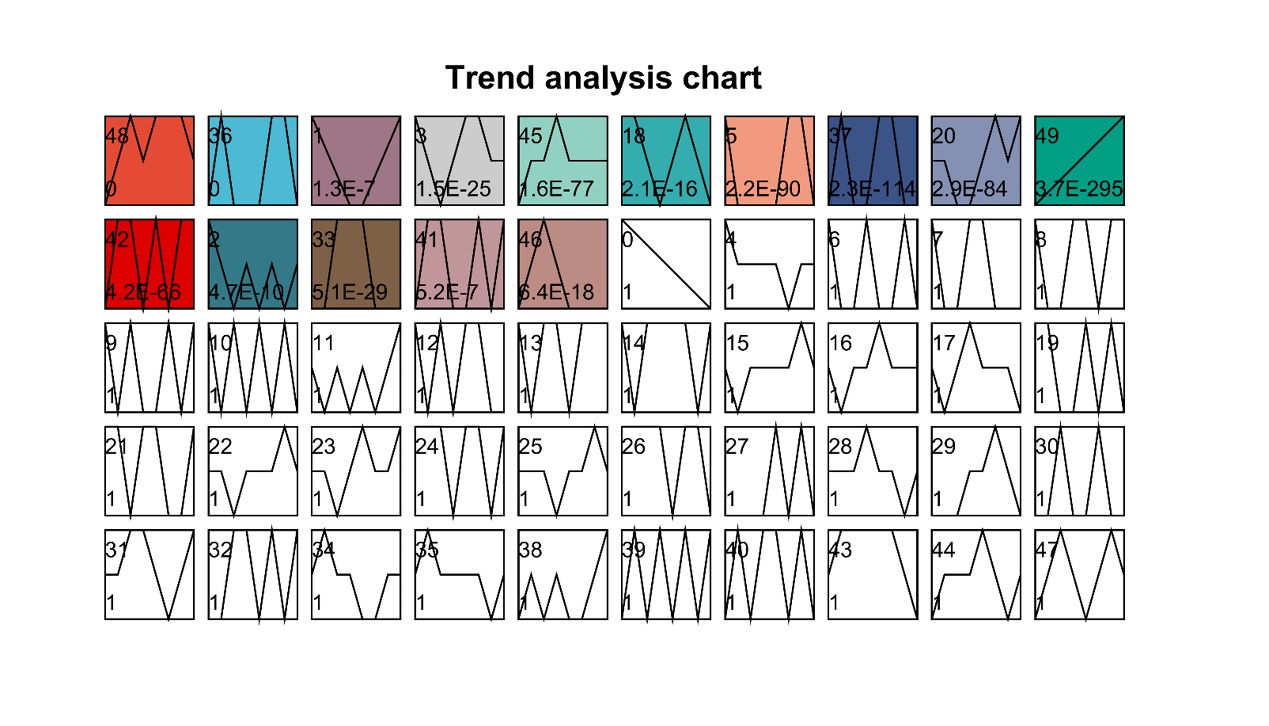


**Fig.S4 Temporal expression trend analysis chart. The number in the upper left corner of the rectangle is the profile number, the broken line is the trend of expression quantity over time, and the value in the lower left corner is its corresponding significance level, p-value. Colored trend charts: indicate that the temporal pattern of the profile is in line with significant change trend.**


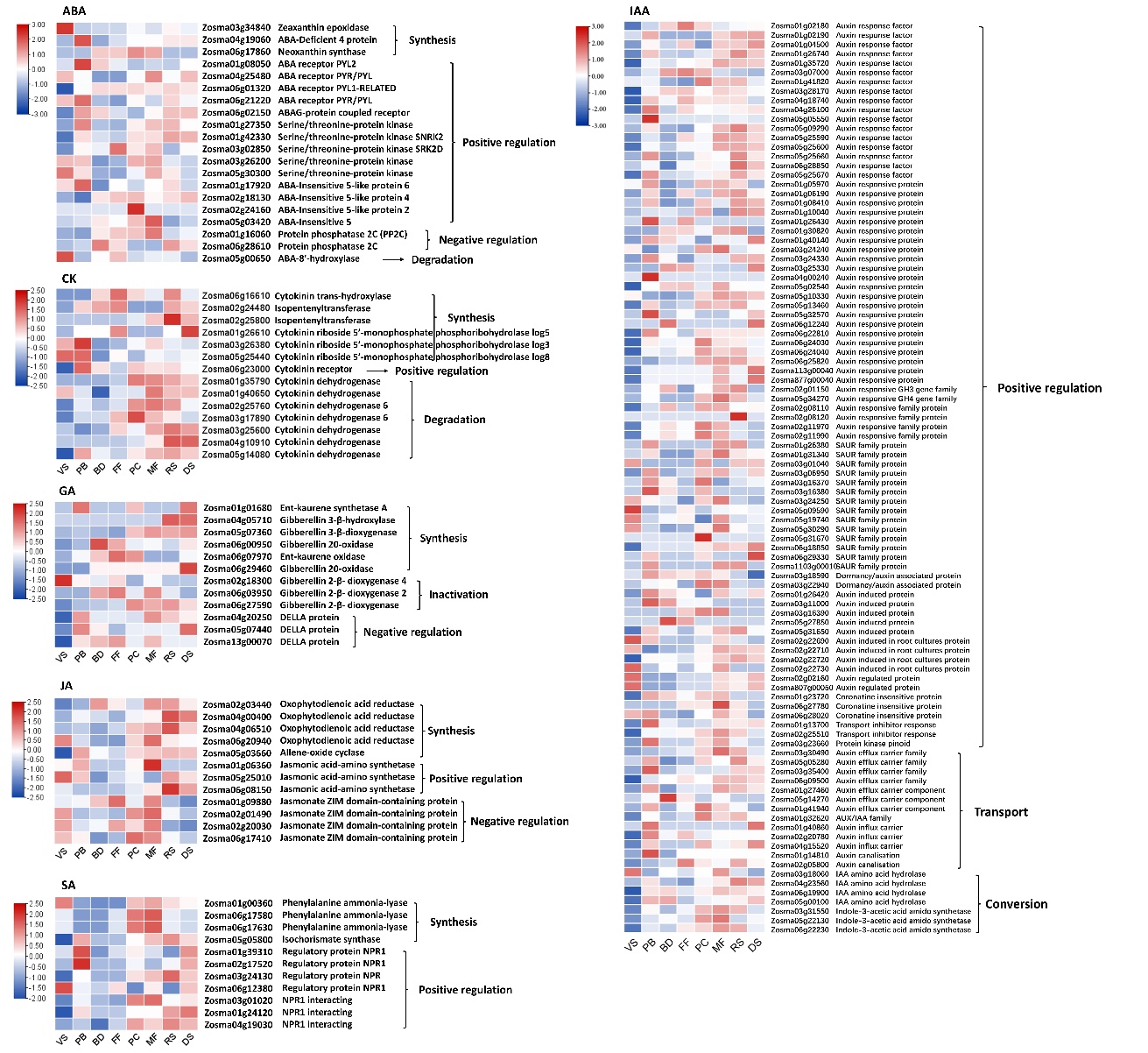


**Fig. S5 Heat map analysis of plant hormones-related genes in eelgrass at various stages. Each column represents average gene expression level of three biological replicates for a specific stage, and each row represents a gene. The color indicates the normalized expression level of the gene in each sample. Red represents higher expression, while blue indicates lower expression. The specific range and trend of expression levels can be found in the numeric scale next to the color bar in the top left corner.**

**Table S1. Primer sequences of RT-qPCR.**

| Num | Gene name | Primer name | Primer sequence（5‘-3’） | Product length |
| --- | --- | --- | --- | --- |
| 1 | Zosma04g26690 | 26690-F | CGACCGCAGTGGTGCTTAC | 249 |
|  |  | 26690-R | CTACCGTTGCCGCCTCTTT |  |
| 2 | Zosma01g34040 | 34040-F | CTGTCAAACCTCCCTGCTT | 297 |
|  |  | 34040-R | ACCGTCGGCTGTCTGTAAT |  |
| 3 | Zosma01g42340 | 42340-F | AACACGGAGGAGAATAGGC | 116 |
|  |  | 42340-R | TCGGGAGTAGATTGGTAGAGT |  |
| 4 | Zosma04g22420 | 22420-F | TCTTGACCCACTTTACCCA | 128 |
|  |  | 22420-R | ATCCGAACATCGAGAACATAG |  |
| 5 | Zosma02g24370 | 24370-F | TAGTTGGGTCGTGATGTTCT | 241 |
|  |  | 24370-R | CATATCTCGGATGACTGTGC |  |
| 6 | Zosma05g29490 | 29490-F | CGATGTTTCGGAGGCTTGT | 278 |
|  |  | 29490-R | CCAATGCTCGGTTGTGGTC |  |
| 7 | Zosma04g09360 | 09360-F | TGCCGTCAACGATCCTTTC | 168 |
|  |  | 09360-R | ATCTCCTCTGGGTTTCTGC |  |
| 8 | Zosma02g18890 | 18890-F | TCCACTACGCTAAGATGAC | 276 |
|  |  | 18890-R | GTCCAGTAGACAGACCCAC |  |
| 9 | 18S rRNA | 18S-F | CAACCATAAACGATGCCGA | 85 |
|  |  | 18S-R | AGCCTTGCGACCATACTCC |  |
